# Evaluation of *In Vitro* Antimalarial Activity of Different Extracts of *Artemisia aucheri* Boiss. and *A. armeniaca* Lam. and Fractions of the Most Potent Extracts

**DOI:** 10.1155/2014/825370

**Published:** 2014-01-14

**Authors:** Mahdi Mojarrab, Ali Shiravand, Abbas Delazar, Fariba Heshmati Afshar

**Affiliations:** ^1^Novel Drug Delivery Research Center, School of Pharmacy, Kermanshah University of Medical Sciences, Kermanshah 67346-67149, Iran; ^2^Student Research Committee, Kermanshah University of Medical Sciences, Kermanshah 67346-67149, Iran; ^3^Drug Applied Research Centre, Tabriz University of Medical Sciences, Tabriz 51656-65811, Iran; ^4^Medical Philosophy and History Research Center, Tabriz University of Medical Sciences, Tabriz 371-51665, Iran

## Abstract

Ten extracts with different polarity from two Iranian *Artemisia* species, *A. armeniaca* Lam. and *A. aucheri* Boiss, were screened for their antimalarial properties by *in vitro* **β**-hematin formation assay. Dichloromethane (DCM) extracts of both plants showed significant antimalarial activities with IC_50_ values of 1.36 ± 0.01 and 1.83 ± 0.03 mg/mL and IC_90_ values of 2.12 ± 0.04 and 2.62 ± 0.09 mg/mL for *A. armeniaca* and *A. aucheri*, respectively. Bioactivity-guided fractionation of DCM extracts of both plants by vacuum liquid chromatography (VLC) over silica gel with solvent mixtures of increasing polarities afforded seven fractions. Two fractions from DCM extract of *A. armeniaca* and four fractions from DCM extract of *A. aucheri* showed potent antimalarial activity with reducing IC_50_ and IC_90_ values compared to extracts. The most potent fraction belonged to DCM extract of *A. armeniaca* with IC_50_ and IC_90_ values of 0.47 ± 0.006 and 0.71 ± 0.006 mg/mL, respectively.

## 1. Introduction

Malaria is one of the most common infectious diseases that are caused by parasites of the genus *Plasmodium* and kills more than one million individuals in the tropical and subtropical zones annually [1, 2]. This situation has been complicated by the appearance of drug-resistant parasites especially to the existing cheap drugs like chloroquine [3]; hence, there has been increasing attempts to identify other alternatives especially plant-derived antimalarial drugs. The genus *Artemisia* (Compositae) is one of the most popular herbs in traditional medicines and mostly used for the treatment of diseases like malaria, hepatitis, cancers, and inflammations [4]. It is a large genus with about 400 species, predominately distributed in the world (especially in Europe, North America, Asia, and South Africa) and 34 species are documented in the flora of Iran [5, 6]. There has been growing attention to this genus since the isolation of Artemisinin, obtained from *A. annua*, and its distinguished clinical effects as a potent antimalarial drug [7]. Artemisinin with an endoperoxide sesquiterpene lactone structure is unlike those of any other known antimalarials (mefloquine, amodiaquine, and chloroquine) that kills all stages of the parasite by a reductive interaction with free heme, resulting in generation of some types of free radicals that could alkylate parasite proteins and damage membranes [8]. Artemisinin also acts by blocking free heme biocrystallization (like 4-aminoquinolines) and hemoglobin degradation [9]. In the last few years, artemisinin and its derivatives were the top of the list of antimalarial drugs against drug-resistant *Plasmodium falciparum* strains, but recently, the emergence of artemisinin-resistant parasites in some regions [10, 11] has led researchers to search for new sources of alternative therapies. In our previous study, dichloromethane extracts of *Artemisia scoparia* and *A. spicigera* were shown to possess antimalarial activity in *β*-hemation formatin assay [12]. As a continuation of our research on Iranian *Artemisia* spp., we have now evaluated antimalarial effect of different extracts and fractions of *Artemisia aucheri* and *A. armeniaca. *In various studies, hypocholesterolemic and antiatherosclerotic effects of *Artemisia aucheri* in rabbits were confirmed [13–16]. Likewise, different extracts of *A. aucheri* have been reported to possess wound healing [17], leishmanicidal [18, 19] and antifungal [20] effects. In the case of *A. armeniaca*, phytochemical evaluations on extracts and essential oil have been carried out and the presence of two new coumarin-hemiterpene ether glycosides [21], four prenylated coumarins, and some known flavonoids [22, 23] was reported. The main constituent of the oil extracted from the aerial parts of *A. armeniaca* was found to be *α*-pinene [24, 25]. The objectives of this study were to investigation of the antimalarial activity of different extracts of these *Artemisia* species (I), fractionation of the most potent extract (II), and determination of the most potent fractions (III).

## 2. Materials and Methods

### 2.1. Chemicals

Hematin porcine, chloroquine diphosphate, sodium dodecyl sulfate (SDS), sodium acetate, magnesium sulfate, sodium hydrogen phosphate, sodium chloride, potassium chloride, sodium hydroxide, glucose, and sodium bicarbonate were purchased from Sigma-Aldrich Chemical Company, oleic acid from Fluka, dimethylsulfoxide, hydrochloric acid, and silica gel 60 (0.040–0.063 mm) from Merck, and all the solvents used for extraction and fractionation from Caledon and Scharlau.

### 2.2. Plant Material

The aerial parts of *Artemisia armeniaca* Lam. were collected from Arasbaran, East Azarbaijan province (Iran), in August 2008. The identity of the plant was confirmed by morphological examination in comparison to the herbarium specimens. Voucher number TBZfph 528 is retained in the School of Pharmacy, Tabriz University of Medical Sciences, Tabriz, Iran. Aerial parts of *Artemisia aucheri* Boiss. were collected from Chahar Bagh region, Golestan province (Iran), in December 2011 and authenticated by Mr. S. A. Hosseini, Agricultural and Natural Resources Research Center of Golestan Province, Gorgan, Iran. A voucher specimen (number 2383) is deposited in the herbarium.

### 2.3. Extract Preparation

The plant samples were air-dried at room temperature under shade, finely ground, and extracted by cold maceration method. 100 g of each plant sample was extracted successively with petroleum ether (PE), dichloromethane (DCM), ethyl acetate (EtOAC), ethanol, and ethanol-water (1 : 1 v/v) at room temperature (sequential maceration with ca. 3 × 1 L of each solvent). All the extracts were separately concentrated using a rotary evaporator at a maximum temperature of 45°C.

### 2.4. Fractionation

DCM extracts of plants (1.54 g of *A. armeniaca* and 1.56 g of *A. aucheri*) were fractionated by vacuum liquid chromatography (VLC) over silica gel (20 g for each) with solvent mixtures of increasing polarities: EtOAC/PE (10 : 90), EtOAC/PE (20 : 80), EtOAC/PE (40 : 60), EtOAC/PE (60 : 40), EtOAC/PE (80 : 20), EtOAC/PE (100 : 0), and methanol. All the fractions were fully dried using a rotary evaporator at a maximum temperature of 45°C.

### 2.5. TLC Analysis of Extracts and Fractions

The identification of main chemical groups was carried out by TLC on silica gel 60 F_254_ Merck (layer thickness 0.25 mm) as follows: for methoxylated flavonoids, chloroform/ethylacetate (60 : 4) and for methylated coumarins, toluene/ether (1 : 1/saturated with 1% acetic acid) were used as solvent system. They were detected under UV 366 nm.

### 2.6. *In Vitro* *β*-Hematin Formation Assay

The potential antimalarial activity of plant extracts was evaluated by the method described by Afshar et al. [12] with some modifications. Briefly, varying concentrations (0–2 mg/mL in DMSO) of the extracts and fractions were incubated with 3 mM of hematin, 10 mM oleic acid, and 1 M HCl. The final volume was adjusted to 1 mL using sodium acetate buffer, pH 5. Chloroquine diphosphate was used as a positive control. The reaction mixtures were incubated overnight at 37°C with constant gentle shaking. After incubation, samples were centrifuged (14,000 rpm, 10 min, at 21°C) and the hemozoin pellet was repeatedly washed with incubation (15 min at 37°C with regular shaking) in 2.5% (w/v) SDS in phosphate buffered saline followed by a final wash in 0.1 M sodium bicarbonate until the supernatant was clear (usually 3–8 washes). After the final wash, the supernatant was removed and the pellets were dissolved in 1 mL of 0.1 M NaOH before determining the hemozoin content by measuring the absorbance at 400 nm (Beckman DU640 spectrophotometer). The results were recorded as %inhibition (*I*%) of heme crystallization compared to negative control (DMSO) using the following equation: *I*% = [(AN − AS)/AN] × 100, where AN: absorbance of negative control and AS is absorbance of test samples.

### 2.7. Statistical Analysis

All experiments were conducted in triplicate measurements and presented as the Mean ± SD. Data were analyzed by Excel 2010 Microsoft. The IC_50_ and IC_90_ values were calculated from nonlinear regression analysis.

## 3. Results

**Figure 1:**
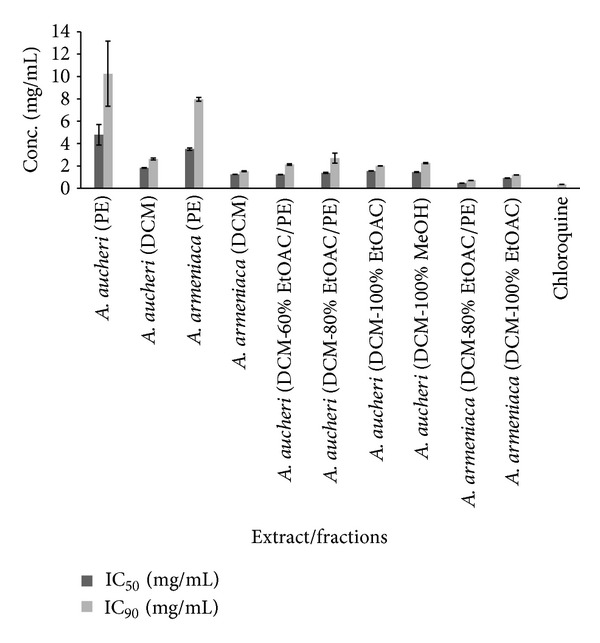
Comparison of IC_50_ and IC_90_ values (mg/mL) of active extracts and fractions of *A. armeniaca* and *A. aucheri* and chloroquine solution in *β*-hematin formation assay. The values were reported as Mean ± SD.

The results from the *in vitro* *β*-hematin formation assay of five different extracts from *A. armeniaca* and *A. aucheri* and seven fractions of their respective DCM extracts as well as the extraction and fractionation yields are listed in Table 1. The inhibition of *β*-hematin formation expressed as percentage (*I*%) and standard deviations (*n* = 3) are given for each extract/fraction. IC_50_ and IC_90_ values were measured graphically by plotting concentrations versus percentage of inhibition. Three extracts (EtOAC, ethanol, and hydroethanol) of both plants had no anti-malarial activity at all while the DCM extracts of *A. armeniaca* and *A. aucheri* compared to the standard anti-malarial compound, chloroquine (IC_50_ = 0.04 ± 0.002, IC_90_ = 0.35 ± 0.006), showed the most potent anti-malarial activity with IC_50_ values of 1.36 ± 0.01 and 1.83 ± 0.03 mg/mL and IC_90_ values of 2.12 ± 0.04, 2.62 ± 0.09 mg/mL, respectively. PE extracts possessed a weak activity with IC_50_ values of 3.51 ± 0.11 and 4.79 ± 0.92 mg/mL and IC_90_ values of 7.96 ± 0.17, 10.25 ± 2.91 mg/mL for* A. armeniaca* and *A. aucheri*, respectively. Among the seven different polarity fractions obtained from the DCM extract of *A. armeniaca*, 80% EtOAC/PE and 100% EtOAC fractions showed considerable anti-malarial activity with IC_50_ values of 0.47 ± 0.006 and 0.94 ± 0.006 mg/mL and IC_90_ values of 0.71 ± 0.006, 1.26 ± 0.02 mg/mL, respectively. In the case of fractions separated from DCM extract of *A. aucheri* four polar fractions (60% EtOAC/PE, 80% EtOAC/PE, 100% EtOAC, and 100% MeOH fractions) showed remarkable anti-malarial effects with close IC_50_ and IC_90_ values (Table 1 and Figure 1).

## 4. Discussion

**Figure 2:**
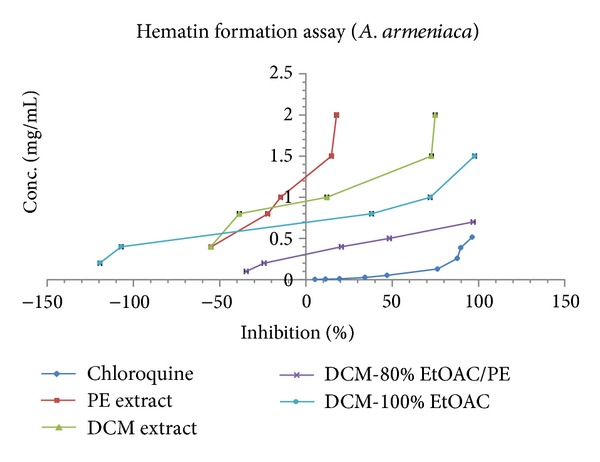
Comparison of % inhibition of heme crystallization between active extracts and fractions of *A. armeniaca* and chloroquine solution in *β*-hematin formation assay. The values were reported as Mean ± SD.

**Figure 3:**
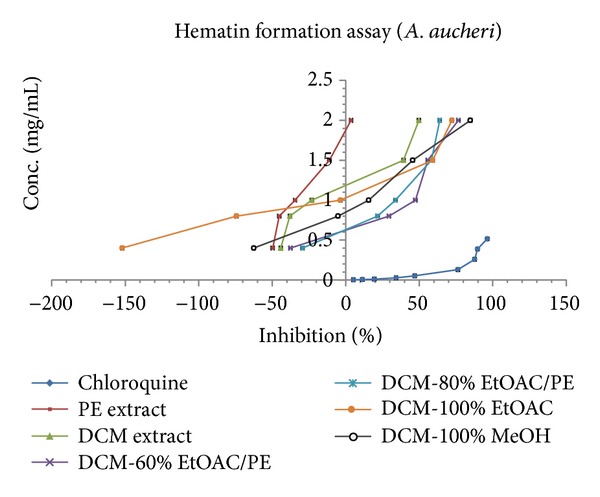
Comparison of %inhibition of heme crystallization between active extracts and fractions of *A. aucheri* and chloroquine solution in *β*-hematin formation assay. The values were reported as Mean ± SD.

The malaria parasite, *Plasmodium*, degrades hemoglobin within the infected erythrocytes to use the catabolic products as the chief source of nutrition for its development and proliferation [26, 27]. Free heme is released as a toxic by-product of this process which could affect cellular metabolism by peroxidizing membranes and inhibiting a variety of enzymes [28]. To protect itself, the malaria parasite uses several detoxification pathways to get rid of excess heme. Polymerization of heme into an insoluble, nontoxic crystalline compound, hemozoin (also called malaria pigment) is believed to be the prominent way of detoxification [29]. Thus, the inhibition of hemozoin formation is an attractive target for development of several antimalarial drugs such as 4-aminoquinolines (quinine, mefloquine, and chloroquine) and is therefore considered as a suitable target for drug screening programs [30]. Many *in vitro* assays based on spectral characteristics and differential solubility of monomeric heme and *β*-hematin (synthetic analogue of hemozoin) have been described and used for screening of novel synthetical [31, 32] and natural [33] antimalarial compounds. In this study, 10 extracts and 14 fractions of *A. armeniaca* and *A. aucheri* were evaluated for their antimalarial activity by an *in vitro* *β*-hematin formation assay developed by Afshar et al. [12]. As shown from the results presented in Table 1 and Figure 1, among the different polarity extracts of *A. armeniaca*, DCM extract showed the most potent activity (IC_50_ = 1.36 ± 0.01 and IC_90_ = 2.12 ± 0.04 mg/mL) and indicated that compounds with strongest antimalarial activity have medium polarity. Subsequent bioactivity-guided fractionation of DCM extract by VLC over silica gel with solvent mixtures of increasing polarities afforded seven fractions. The activity of 80% EtOAC/PE and 100% EtOAC fractions was observed to be significantly higher than that of DCM extract and 80% EtOAC/PE fraction was determined the most active fraction. The inhibitory activity of these two fractions was comparable with that of standard drug chloroquine (IC_50_ = 0.04 ± 0.002 and IC_90_ = 0.35 ± 0.006 mg/mL) while the remaining fractions were considered inactive. TLC analysis of the DCM extract and its fractions indicated the presence of terpenes, fatty acids, methylated coumarins, and methoxylated flavonoids as major constituents. Previous investigations showed that methoxylated flavonoids [34], terpens, steroids [35], saponins [36], and methylated coumarins [37] exhibited antiplasmodial activity in different antimalarial assays. In 80% EtOAC/PE and 100% EtOAC fractions, methoxylated flavonoids and methylated coumarins were identified by TLC analysis and could be considered as the major active constituents. Therefore, it seems that the potent antimalarial activity of *A. armeniaca* DCM extract and its active fractions might be related to the presence of these compounds. As observed in Table 1, similar findings were illustrated in case of *A. aucheri* extracts and fractions. DCM extract was found to be more potent (IC_50_ = 1.83 ± 0.03, IC_90_= 2.62 ± 0.09 mg/mL) than the corresponding PE extract (IC_50_ = 4.79 ± 0.92, IC_90_ = 10.25 ± 2.91 mg/mL) while the other three polar extracts were inactive. The DCM extract was selected for further investigation because of its potent activity and subjected to fractionation by the above-mentioned procedure. Among the seven different polarity fractions, 10% EtOAC/PE, 20% EtOAC/PE, and 40% EtOAC/PE fractions revealed no activity in this assay system, while the last four polar fractions showed the potent activity with close IC_50_ and IC_90_ values (Table 1). Methoxylated flavonoids and methylated coumarins were identified by TLC analysis from 60% EtOAC/PE, 80% EtOAC/PE, 100% EtOAC, and 100% MeOH fractions. Furthermore, in previous phytochemical study, two endoperoxide derivatives were isolated from the aerial parts of *A. aucheri *[38] that structurally similar to artemisinin. Additionally, in recent study, the presence of amorpha-4,11-diene synthase, a key enzyme in artemisinin production, was demonstrated in *A. aucheri* [39]. Therefore, the possibility that the antimalarial activity displayed by DCM extract and its active fractions reported here would be due to the presence of these types of compounds could not be excluded. Overall, the results from this investigation showed that both DCM extracts of these *Artemisia* species act as inhibitors of heme crystallization pathway and *A. armeniaca* illustrated more potent activity than *A. aucheri*. A comparison of DCM fractions of two tested plants based on IC_50_ and IC_90_ values (Figure 1) revealed that 80% EtOAC/PE fraction of *A. armeniaca* were about 3-fold and 4-fold more potent than 80% EtOAC/PE fraction of *A. aucheri*, respectively. This difference might have been derived from the high concentration of antimalarial components in this fraction and removing as much the lipid-like compounds from it. As shown in Figures 2 and 3, at lower concentrations of the active extracts and fractions, the observed absorbance was higher than the negative control which might be due to the presence of other fatty acids and lipids causing synergistic effect with oleic acid in the assay. It was demonstrated that the IC_50_ and IC_90_ values could be reduced by completely removing the lipids and purifying the active antimalarial principles.

## 5. Conclusion

From the selection of 10 extracts with different polarity, results showed that the DCM extract of *A. armeniaca* was the most active extract in *β*-hematin formation assay followed by the DCM extract of *A. aucheri*. This preliminary study and its data persuade us to focus on purifying the active components of these extracts and investigating further on animal models for *in vivo* evaluation.

## Figures and Tables

**Table 1:** The 50% and 90% inhibition concentrations (mg/mL) of different extracts and fractions of *A. armeniaca* and *A. aucheri* in *β*-hematin formation assay.

Plants	Extracts/fractions	Yields (%)	IC_50_ (mg/mL)^a^	IC_90_ (mg/mL)^a^
*Artemisia aucheri* (aerial parts)	Petroleum ether	1.11	4.79 ± 0.92	10.25 ± 2.91
	Dichloromethane	4.46	1.83 ± 0.03	2.62 ± 0.09
	Ethyl acetate	0.46	—	—
	Ethanol	9.09	—	—
	Ethanol-water	7.27	—	—

*Artemisia armeniaca* (aerial parts)	Petroleum ether	1.88	3.51 ± 0.11	7.96 ± 0.17
	Dichloromethane	1.97	1.36 ± 0.01	2.12 ± 0.04
	Ethyl acetate	0.56	—	—
	Ethanol	2.57	—	—
	Ethanol-water	17.66	—	—

*Artemisia aucheri* (DCM fractions)	10% EtOAC/PE	16.86	—	—
	20% EtOAC/PE	8.08	—	—
	40% EtOAC/PE	24.68	—	—
	60% EtOAC/PE	15.06	1.23 ± 0.01	2.13 ± 0.07
	80% EtOAC/PE	7.12	1.38 ± 0.05	2.70 ± 0.44
	100% EtOAC	8.91	1.55 ± 0.01	2.01 ± 0.01
	100% Methanol	10.51	1.45 ± 0.03	2.26 ± 0.05

*Artemisia armeniaca* (DCM fractions)	10% EtOAC/PE	3.25	—	—
	20% EtOAC/PE	3.90	—	—
	40% EtOAC/PE	13.88	—	—
	60% EtOAC/PE	7.48	—	—
	80% EtOAC/PE	15.84	0.47 ± 0.006	0.71 ± 0.006
	100% EtOAC	6.49	0.94 ± 0.006	1.26 ± 0.02
	100% Methanol	40.91	—	—

Chloroquine	—	—	0.04 ± 0.002	0.35 ± 0.006

^a^Experiment was performed in triplicate and expressed as Mean ± SD.
